# Removal of Arsenate by Fixed-Bed Columns Using Chitosan-Magnetite Hydrogel Beads and Chitosan Hydrogel Beads: Effect of the Operating Conditions on Column Efficiency

**DOI:** 10.3390/gels9100825

**Published:** 2023-10-19

**Authors:** Eduardo Mendizabal, Nely Ríos-Donato, Carlos Federico Jasso-Gastinel, Ilse Paulina Verduzco-Navarro

**Affiliations:** 1 Chemistry Department, CUCEI, University of Guadalajara, Blvd. Gral. Marcelino García Barragán 1421, Guadalajara 44430, Jalisco, Mexico; eduardo.mmijares@academicos.udg.mx (E.M.); nely.rios@academicos.udg.mx (N.R.-D.); 2 Chemical Engineering Department, CUCEI, University of Guadalajara, Blvd. Gral. Marcelino García Barragán 1421, Guadalajara 44430, Jalisco, Mexico; carlos.jasso@academicos.udg.mx

**Keywords:** adsorption, arsenate, fixed-bed, chitosan, magnetite

## Abstract

Fixed-bed columns packed with chitosan-magnetite (ChM) hydrogel and chitosan (Ch) hydrogel were used for the removal of arsenate ions from aqueous solutions at a pH of 7.0. The effect of flow rate (13, 20, and 25 mL/h), height of the columns (13 and 33 cm), and initial arsenate concentration (2, 5 and 10 mg/L) on the column’s efficiency for the removal of As(V) is reported. The maximum adsorption capacity (q_b_), obtained before the allowed concentration of contaminant is exceeded, the adsorption capacity (q_e_) when the column is exhausted, and the mass transfer zone were determined. With this information, the efficiency of the column was calculated, which is given by the H_L_/H_LUB_ ratio. The higher this ratio, the higher the efficiency of the column. The highest efficiency and the highest uptake capacity value at breakthrough point were obtained when using the lower flow rate, lower initial arsenate concentration, and longer bed length. When 33 cm-high columns were fed with a 10 mg As(V)/L solution at 13 mL/h, the maximum uptake capacity values at exhaustion obtained for Ch and ChM were 1.24 and 3.84 mg/g, respectively. A pH increase of the solution at the column’s exit was observed and is attributed to the proton transfer from the aqueous solution to the amino and hydroxyl groups of chitosan. The incorporation of magnetite into Ch hydrogels significantly increases their capacity to remove As(V) due to the formation of complexes between arsenic and the magnetite surface. Experimental data were fitted to the Thomas model, the Yoon–Nelson model and the Bohart–Adams model using non-linear regression analysis.

## 1. Introduction

The availability of good-quality water is necessary for the sustenance of life; the contamination of water bodies with hazardous components causes severe detrimental effects on the health of its consumers. According to the World Health Organization, the ingestion of water with arsenic concentration above 10 μg/L augments human mortality due to its capacity to cause various cancer types [1]. Arsenic concentrations up to 2000 µg/L have been reported in Latin American drinking waters [2]. In Mexico, drinking waters with up to 1004 µg /L of arsenic [3] and superficial waters used for irrigation with up to 8684 µg As/L have been detected [4]. Arsenic contamination may originate from anthropogenic sources derived from activities such as burning fossil fuels, mining, smelting, and using agrochemicals containing As, among others [5,6]. Arsenic present in water is mainly in the form of the inorganic oxoanions arsenite (AsO_3_^−^) and arsenate (AsO_4_^3−^) [7,8], and under oxidizing conditions, the pentavalent for of arsenic is prevalent [8,9]. The pH values in most natural waters usually lie in the range of 6–9 [6,10], so the predominant species of As(V) are anionic species H_2_AsO_4_^−^ and HAsO_4_^2−^ [11]. There are several technologies for the elimination of metal ions from water, such as: chemical coagulation, biological treatment, electrochemical oxidation, ozonation, ultrafiltration, and adsorption [12]. Adsorption is a widely used method due to its simple design and applicability, and because its low operation costs [13,14]. Although activated carbon is a quite effective universal adsorbent, its widespread use in water treatment is limited due to its high cost; this is why adsorption using adsorbents obtained from agro-industrial wastes is a cost-effective alternative method for the removal of contaminants from water [15].

For large-scale water treatment, continuous flow operations with packed columns are preferable over batch systems because they are simpler to operate, have a greater removal efficiency and can treat large volumes of water [16,17]. When an adsorption process is operated using a batch system, the solution’s final concentration cannot be lower than the equilibrium concentration; in contrast, when operating the process using packed columns, the effluent concentration at the column’s exit can be zero or considerably small for a long operation time, which results in the obtention of a large volume of highly-purified solution [18,19].

Chitin is the second-most-abundant naturally occurring form of polymerized carbon, and it is obtained from seafood industry waste [20,21]. The partial deacetylation of chitin results in the obtention of chitosan (Ch), which is a biopolymer reported to be effective as an adsorbent for the removal of water contaminants, due to the presence of amine and hydroxyl groups in its structure which are capable of interacting with ionic species [22,23]. Chitosan is supplied in the form of powder or flakes, but its direct use for packing columns for large-scale water treatment is disadvantageous because it can cause a significant drop in hydrodynamic pressure or clogging of the column [17,24,25]. Therefore, chitosan is used to pack columns as hydrogel beads.

Chitosan can be used as a polymeric matrix to incorporate other adsorbent materials for the obtention of composites with improved functional properties [26]. Iron oxides are considered to be useful as arsenic adsorbents since they are capable of forming complexes with arsenic oxyanions and they are considered to be low-cost materials [27]. Particularly, magnetite Fe_3_O_4_ nanoparticles are recognized as adsorbents for heavy metal ions and metalloids due to their large surface area and high reactivity [28]. Additionally, iron oxides present a high affinity for chitosan [1,29]; chitosan-iron oxide composites have been used for the treatment of arsenic-contaminated aqueous systems [30,31,32]. Arsenate has been removed from aqueous systems at pH 7 using chitosan-magnetite (ChM) hydrogel beads in batch systems, reporting isotherm data best being fitted by Langmuir model and pseudo-first order kinetics [33].

The present work reports the removal of arsenate ions from aqueous solutions at a pH of 7.0 using fixed-bed columns packed with ChM and Ch hydrogel beads, looking for efficient conditions for the adsorption process. The effect of the initial As(V) concentration, flow rate, and column height on the shape of the breakthrough curves, the As(V) removal capacity at the breakthrough point and the exhaustion point, as well as the effect of the experimental conditions on the development of the mass transfer zone (MTZ) and the efficiency of the process are presented. Experimental data fitting to three fixed-bed models: the Thomas model, Yoon–Nelson model, and Adams-Bohart model, was performed using non-linear regression analysis.

## 2. Results and Discussion

### 2.1. Characterization of the Hydrogel Beads

#### 2.1.1. Hydrogel Beads Composition

By gravimetry, it was obtained that ChM hydrogel contained 93.5 ± 0.1% water and Ch hydrogel contained 92.0 ± 0.4% water. The ash content of the ChM xerogel beads was 10.3 ± 0.4%, and that of the Ch xerogel was 1.3 ± 0.1% ashes. Therefore, it was determined by Equation (2) that the magnetite content of the ChM hydrogel beads was 0.6 ± 0.1% and by Equation (3) that the Ch content in the ChM hydrogel was 5.9 ± 0.5% and 8.0 ± 0.4% in the Ch hydrogel.

#### 2.1.2. Average Diameter of the Hydrogel Beads

A sample of 30 ChM hydrogel beads was measured, resulting in the average diameter of the beads being 2.55 ± 0.03 mm. The average diameter of a sample of 30 Ch hydrogel beads was 2.27 ± 0.13 mm.

#### 2.1.3. Characterization of the ChM Hydrogel Beads

**Figure 1:**
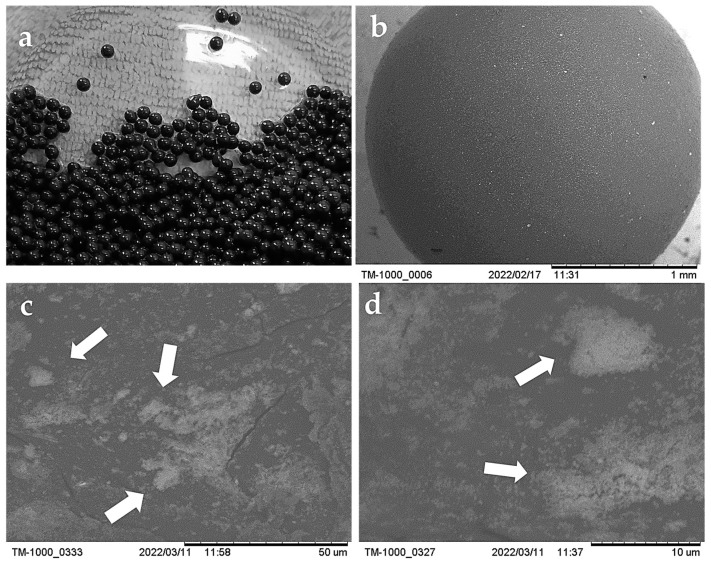
Images of ChM beads obtained (**a**) with a digital camera; (**b**–**d**) with a scanning electron microscope (SEM), where magnetite particles are signaled with arrows.

Figure 1 shows that the ChM hydrogel beads are spherical (Figure 1a,b) with an irregular surface (Figure 1c). It can be observed that there is a good dispersion of the magnetite nanoparticles in the hydrogel beads (Figure 1b) and that, in some regions, these nanoparticles form clusters (Figure 1c,d).

#### 2.1.4. Potentiometric Titration

The pKa values of Ch and ChM were obtained from the titration data and by linear regression analysis using the Henderson–Hasselbalch equation [34], obtaining a pKa value of 6.04 ± 0.03 for Ch and 6.16 ± 0.01 for ChM.

#### 2.1.5. Interaction of Ch and Magnetite Determined by FTIR

**Figure 2:**
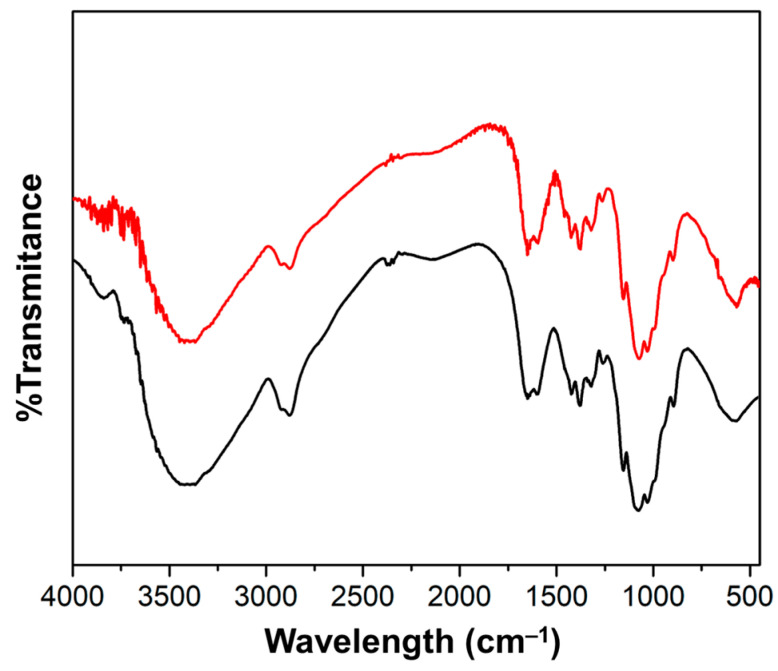
FTIR spectra of (**—**) Ch and (**—**) ChM.

Figure 2 shows the characteristic signals of Ch where O–H flexions can be observed in the broad band around 3422 cm^−1^ and at 1380 cm^−1^; the N–H flexion signal is also observed at 3422 cm^−1^ band and at 1650 cm^−1^. Figure also shows the FTIR spectrum of ChM, where C–O signals shifted from 1380 to 1376 cm^−1^ and from 1077 to 1073 cm^−1^ when magnetite was incorporated into the Ch hydrogel.

#### 2.1.6. Interaction of ChM and Arsenate Determined by XPS

**Figure 3:**
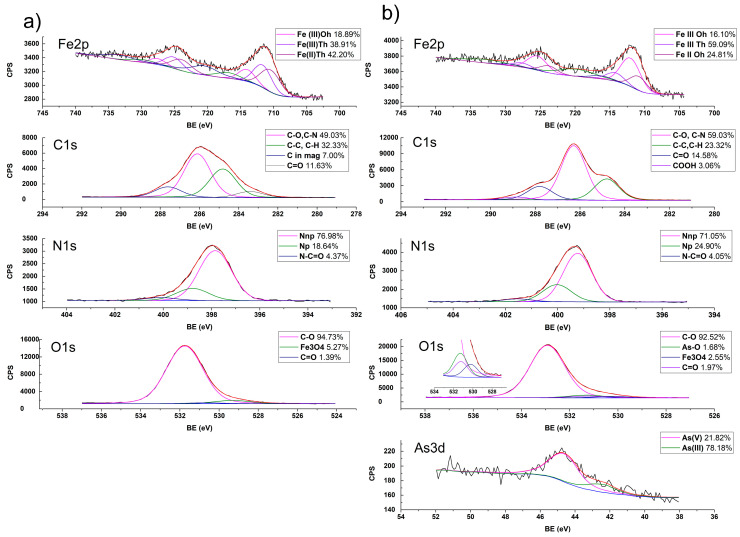
XPS spectra of ChM (**a**) before and (**b**) after arsenate adsorption.

High-resolution spectra of ChM composite, before and after the adsorption of arsenate, were obtained by XPS. In the C1s region after deconvolution (Figure 3a) four signals for the chitosan were observed: the signal at 287.6 eV corresponded to the C=O bonds, the 286.1 eV signal was due to C–O and C–N bonds, while the 284.8 eV signal is due to the C–C and C–H bonds [35,36,37] and the fourth signal al 283.4 eV is attributed to carbon absorbed on magnetite [38]. Three signals were obtained in the O1s region: the most significant signal at 531.7 eV was due to C–O bonds, the signal at 530.7 eV corresponded to C=O bonds, and the 529.3 eV signal was due to oxygen atoms in magnetite [38,39,40]. Three signals were obtained in the N1s region: the first at 400.1 eV belonged to the amide group, and those at 399.0 and 397.8 eV corresponded to the amine group in its protonated and unprotonated form. The values obtained were lower than the values reported for chitosan [28,31], because of the amine groups interacting with magnetite. In the Fe2p region, three signals were obtained: the signal at 710.4 eV was for the tetrahedral Fe(II) ions (Th), the signal at 711.6 eV was due to the tetrahedral Fe(III) ions, and the signal at 714.0 eV corresponded to the octahedral Fe(III) ions. A value of 2:2:1 was obtained for the ratio Fe(II)_Th_:Fe(III)_Th_:Fe(III)_Oh_ in ChM, which differs from the value reported for magnetite (1:1:1:1) [31], which is attributed to the interaction of iron ions with chitosan.

Figure 3b shows the XPS spectra of ChM with adsorbed arsenic. In the C1s region, a new signal is observed at 288.9 eV, corresponding to protonated carbonyl groups, and the four signals observed in ChM remain at the same binding energies.

At the N1s region, a shift is observed in binding energies, indicating that nitrogen atoms interact with arsenic during adsorption. A shift of the binding energies in the O1s region is observed, indicating the interaction of C-O and C=O with arsenic, and a signal appears at 531.3 eV due to As binding to the O of the arsenate [40].

After arsenic adsorption, the signals in the Fe2p region remained unchanged. However, their intensities were modified, yielding a Fe(II)_Th_:Fe(III)_Th_:Fe(III)_Oh_ ratio of 1.3:3.7:1, indicating that a part of the Fe(II) ions were oxidized to Fe(III) ions. Figure 2b shows the As3d region, where two signals appear at 44.37 and 42.41 eV, attributed to As(V) and As(III), respectively. The presence of As(III) is due to the reduction of As(V) when Fe(II) ions in magnetite were oxidized to Fe(III) ions [33,38]. It has been reported that in the formation of the ChM-As complex, a portion of As(V) is reduced to As(III) as some Fe(II) ions in magnetite are oxidized to Fe(III) ions [40,41].

### 2.2. Arsenate Adsorption Studies in Fixed-Bed Columns

Table 1 shows the following results: the amount of arsenate removed by the ChM or Ch hydrogel beads (mg of As(V)/g of xerogel) at the breakthrough point (q_b_) and at the end of the column operation (q_e_), the length of the unused bed length (H_LUB_), efficiency expressed as the ratio of bed used H_L_ and unused bed H_LUB_ (H_L_/H_LUB_), the treated volume (mL) at breakthrough, the number of interstitial bed volumes (NIVB) that have passed through the columns at the time of breakthrough (t_b_) and at the exhaustion of the column (t_e_). The breakthrough point was chosen when the arsenate concentration of the effluent (C_t_) reached 5% of the concentration of feed solution (C_0_).

**Figure 4:**
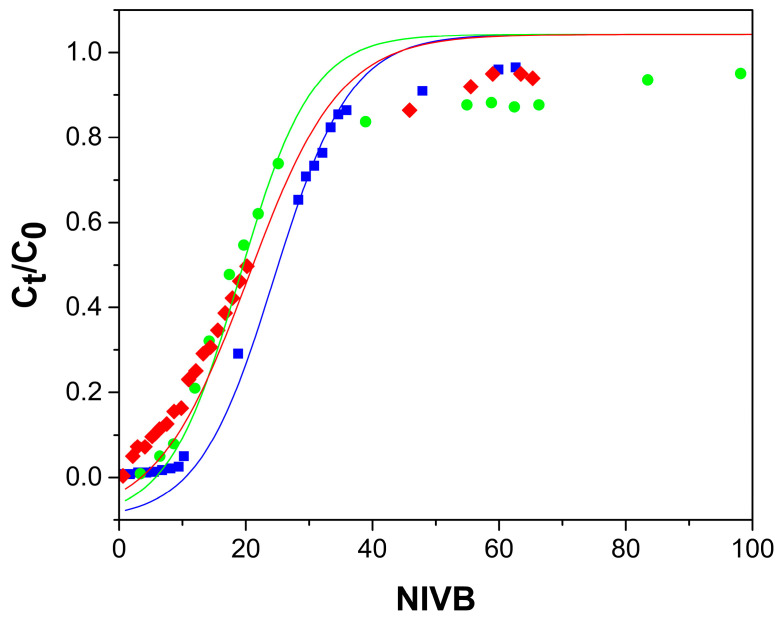
Breakthrough curves for the removal of As(V) by ChM hydrogel beads, column height 13 cm, and As(V) concentration of 5 mg/L. Experimental data for flow rate of 13 mL/h (■), 20 mL/h (●), and 25 mL/h (♦). Thomas model predictions (—).

**Figure 5:**
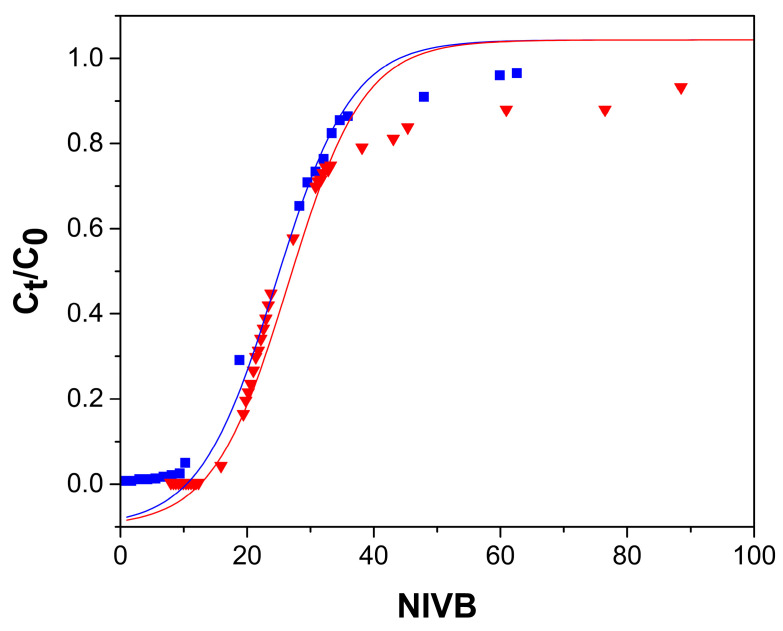
Breakthrough curves for the removal of As(V) by ChM hydrogel beads; flow rate of 13 mL/h, and As(V) concentration of 5 mg/L. Experimental data for bed height of 13 cm (■) and 33 cm (▼). Thomas model predictions (—).

**Figure 6:**
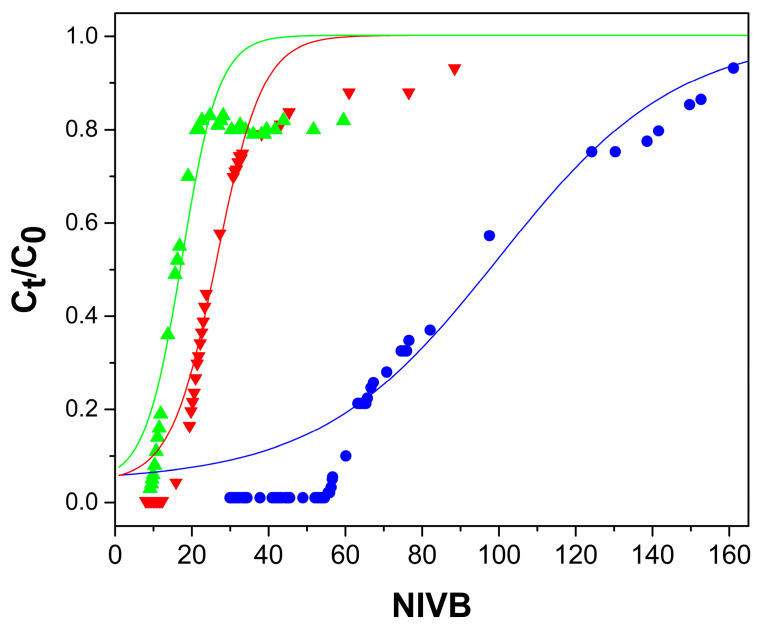
Breakthrough curves for the removal of As(V) by ChM hydrogel beads, column height 33 cm, and flow rate of 13 mL/h. Experimental data for As(V) concentrations of 2 mg/L (●), 5 mg/L (▼), and 10 mg/L (▲). Thomas model predictions (—).

**Figure 7:**
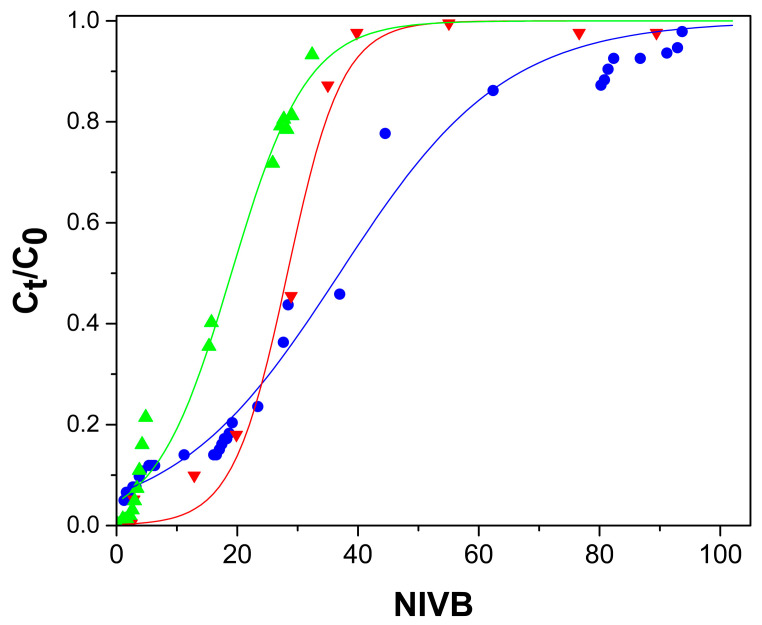
Breakthrough curves for the removal of As(V) by Ch hydrogel beads, column height 33 cm, and flow rate of 13 mL/h. Experimental data for As(V) concentrations of 2 mg/L (●), 5 mg/L (▼), and 10 mg/L (▲)). Thomas model predictions (—).

Figure 4, Figure 5, Figure 6 and Figure 7 show the breakthrough curves obtained by plotting C_t_/C_0_ as a function of the NIVB. These curves show the characteristic sigmoidal shape [16,18,19] obtained in adsorption processes operated in continuous mode using fixed-bed columns.

#### 2.2.1. Effect of Flow Rate

The effect of the flow rate was determined by comparing the removal of As(V) from a 5 mg As(V)/L aqueous solution at pH 7.0 using three flow rates (13, 20 and 25 mL/h) in a 13 cm high column packed with ChM beads. Figure 4 shows the breakthrough curves at the three flow rates, where it can be observed that fewer NIVB passed through the columns at the breakthrough concentration and that the curves became less steep when the flow rate was increased. Table 1 reports that by increasing the flow rate, a decrease in q_b_ is observed and q_e_ was practically unmodified. The axial dispersion of the adsorbate, the external resistance of the film, and the intraparticle diffusion resistance cause the adsorbate to need more time in the column to reach the active sites of the hydrogel beads [16,42,43]. When the flow rate becomes higher, the residence time of the As(V) decreases, so q_b_ is decreased. However, when axial dispersion and mass transfer resistances are small, the breakthrough curve will behave close to a step function, and the breakthrough curves will be practically velocity independent [18,44].

The efficiency of the adsorption process was evaluated by the ratio of H_L_ (the amount of used adsorbent) and H_LUB_ (which represents the MTZ). Table 1 shows that as the flow rate increases, the MTZ increases and the H_L_/H_LUB_ ratio decreases. At the three flow rates, the amount of adsorbent used to remove As(V) is much less than the unused, which resulted in H_L_/H_LUB_ values smaller than 1.0, meaning that the MTZ had not reached equilibrium, so the columns are not considered to be efficient [19].

#### 2.2.2. Bed Height Effect

The effect of bed height was determined by comparing the As(V) removal capacity of a 13 cm high column with a 33 cm high column. Both columns were fed with aqueous As(V) solutions with a pH of 7.0, a concentration of 5 mg As(V)/L and a flow rate of 13.0 mL/h. Figure 5 shows that by increasing bed height, the NIVB obtained when breakthrough was reached increased, and that the curve became steeper. Table 1 shows that, by increasing bed height from 13 to 33 cm, the NIVB at breakthrough increased by approximately 50%, the quantity of As(V) removed at breakthrough (q_b_) is about five times larger, and at q_e_, is three times larger. The increase in q_b_ and q_e_ can be attributed to the increased number of active sites in the bed available to interact with the solute previous to the solution exiting the column. It has been reported that a larger q_b_ is obtained when increasing bed height [19,45]. In Table 1, it can be observed that with an increase in bed length, the amount of adsorbent used for As(V) removal and the ratio H_L_/H_LUB_ are larger, indicating that the removal efficiency of the column increased. The process is considered efficient when the MTZ (H_LUB_) is equal or less than H_L_ [18,46]; therefore, when the bed length was increased to 33 cm, the columns were efficient, since H_L_/H_LUB_ values greater than 1 were obtained.

#### 2.2.3. As(V) Concentration Effect

The effect of the initial As(V) concentration was determined by using three different concentrations: 2, 5 and 10 mg As(V)/L, a column with 33 cm height packed with ChM hydrogel beads and a flow rate of 13 mL/h. Figure 6 shows that with increasing As(V) concentration, lower NIVB are obtained at the breakthrough time (t_b_) and column exhaustion time (t_e_) and steeper curves were obtained. It has been previously reported that steeper breakthrough curves are obtained when larger adsorbate concentration is used because the time to reach the breakthrough point and the exhaustion of the column is reduced [47,48]. Table 1 shows that NIVB and the H_LUB_/H_L_ ratio decreased when the initial As(V) concentration was increased, but q_b_ was practically unchanged, and that the H_L_/H_LUB_ ratio was higher than 1.0 at the three concentrations used, indicating that the MTZ was fully developed, and the removal processes were efficient.

The effect of the initial As(V) concentration when using the Ch hydrogel beads was determined using the same concentrations, columns, and flow rate used in the ChM hydrogel beads experiments. Figure 7 shows that the shape of the breakthrough curves obtained with the column packed with Ch are similar to that packed with ChM. Table 1 shows that for the column packed with Ch, q_b_, q_e_ and H_L_/H_LUB_ ratio increased with increasing As(V) concentration; however, they were much lower than the values obtained with the ChM-packed column. The values obtained for H_L_/H_LUB_ ratio were well below 1.0, indicating that the MTZ had not been fully developed, so the columns did not efficiently remove As(V).

The highest q_e_ value obtained when using the Ch hydrogel was 1.24 ± 0.09 mg As(V)/g, which is much lower than the q_e_ value (3.84 ± 0.12 mg As/V)/g) obtained with the ChM hydrogel under the same experimental conditions; therefore, incorporating magnetite into the Ch hydrogel is required to obtain a higher removal capacity. The value obtained for q_e_ using the ChM hydrogel is higher than other q_e_ values reported for As(V) removal at pH near 7 using fixed beds packed with other iron-containing adsorbents (Table 2).

#### 2.2.4. pH Effect

**Figure 8:**
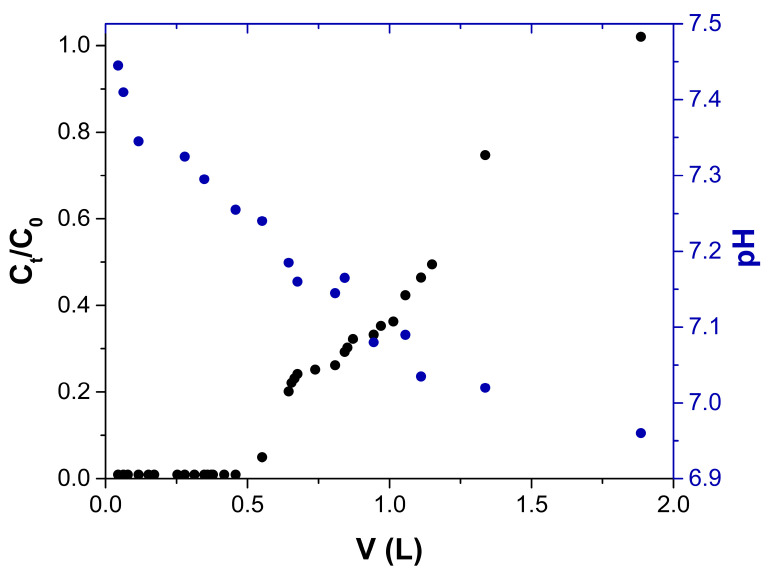
Breakthrough curve (●) and effluent pH (●) in the removal of As(V) using a fixed-bed column packed with the ChM hydrogel beads, feeding a solution with 5 mg As(V) with a pH of 7.0.

The pH of the effluent was measured during the operation of the columns. Figure 8 shows the exiting aqueous solution’s pH and C_t_/C_0_ as a function of the treated volume. Initially, at the column’s exit, the solution’s pH was higher (7.45) than that of the inlet (6.98) and then decreased to a pH of 6.96 at the end of the column operation. The increase in effluent pH is due to the protonation of the chitosan functional groups, mainly the amino groups, by the transfer of protons from the aqueous solution, which is favorable for the adsorption process since protonated amino and hydroxyl groups can interact with arsenic oxyanions [32,33,58].

**Figure 9:**
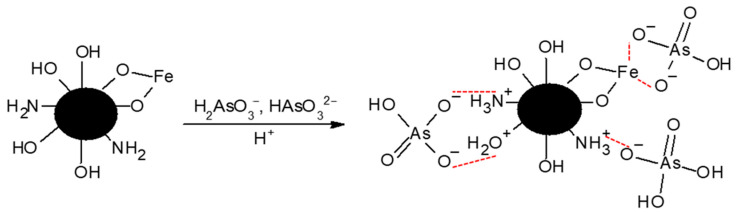
Arsenate adsorption mechanism onto ChM hydrogel.

In Figure 9, the proposed arsenate adsorption mechanism onto ChM is presented. At the pH of 7, H_2_AsO_4_^−^ and HAsO_3_^2−^ are the two predominant species of arsenate present [59,60]. According to the Henderson–Hasselbach equation (Equation (4)), at 7.0, approximately 12.63% of chitosan’s amine groups in ChM are protonated and are capable of interacting with these arsenic oxyanions. When ChM is in an aqueous medium, some of the hydroxyl groups of chitosan and hydrated magnetite are protonated and can interact with arsenate ions [32]. When considering the crystal structure of magnetite, Fe(II) and Fe(III) ions present on the surface of the mineral are capable of interacting with oxygen atoms from the arsenic oxyanionic species, allowing the formation of complexes during the adsorption process [41,61]. Magnetite has been previously reported to have a high arsenic removal capacity (3.4 mg/g at pH 6), and that when the pH increases from 6 to 7, the adsorption of As(V) by magnetite decreases only slightly [40]. Therefore, magnetite is responsible for most of the arsenate removal at the pH used in this study (7.0).

### 2.3. Modelling of the Behavior of the Fixed-Bed Column

Experimental data were fitted to the Thomas model (Equation (9)), the Yoon–Nelson model (Equation (10)), and the Bohart–Adams model (Equation (11)). The parameters obtained for these models by non-linear regression analysis are shown in Table 3. The Thomas model and Yoon–Nelson model both adequately described the columns’ behavior with the same correlation coefficient (R^2^), obtaining high values for the adjustment parameter and low values for the mean average error (MAE). However, considering that our adsorption system is liquid-solid and that it has been reported that in arsenate removal using ChM hydrogel beads, the adsorption followed a Langmuir isotherm [26], the Thomas model was considered more suitable for the description of these columns.

For data obtained using the ChM hydrogel, a good correspondence is observed between the experimental data and the values predicted by the Thomas model, up to values around C_t_/C_0_ of 0.8, (Figure 4, Figure 5 and Figure 6). Past this point, the model predicts higher values than those obtained experimentally; these differences are due because part of the adsorbed As(V) is reduced to As(III) by the ferric ions of the magnetite. Fe(II) ions in magnetite have been reported to reduce As(V) to As(III) [33,41]. The predominant As(III) species at pH (7.0–7.4) of the adsorption process is the neutral species H_3_AsO_3_, which cannot interact with the active sites of the adsorbent [33,59] and then desorbs, leaving those sites ready to adsorb As(V). When using Ch hydrogel beads (Figure 7), there is a good correspondence between the data and predicted values, even past the point of C_t_/C_0_ = 0.8, which corroborates that magnetite is causing the discrepancy between the experimental data and predicted values above C_t_/C_0_ = 0.8.

## 3. Conclusions

ChM hydrogel beads with an average diameter of 2.55 mm were obtained with good dispersion of the magnetite nanoparticles. FTIR and XPS analyses indicate that amine and hydroxyl groups of Ch interact with magnetite in the ChM composite. ChM interacts with arsenate species through protonated amine and hydroxyl groups of Ch and forming complexes with iron ions from magnetite.

Arsenate adsorption onto ChM hydrogel beads was studied using fixed-bed columns at near-neutral pH. When the flow rate was decreased, the column efficiency increased because the contact time between arsenate and adsorbent increased; when the column height was increased, q_b_ increased significantly, and H_L_/H_LUB_ values greater than 1.0 were obtained, indicating a high efficiency of column operation. The full development of the MTZ was only obtained when 33 cm high columns were used. With increasing As(V) concentration in the feed, q_b_ remained practically unchanged. However, when using the Ch hydrogel, the q_b_ value is augmented and their values were much smaller than those obtained with the ChM hydrogel; the full development of MTZ could not be obtained. Incorporating magnetite into Ch hydrogels significantly increases their capacity to remove As(V). The pH of the effluent solution increased initially, but during the adsorption process, it decreased, and at the end of the column operation, the pH was that of the inflow. The protonation of amine and hydroxyl groups in Ch was favorable for removing arsenic oxyanionic species at the studied pH.

For column scaling, it is necessary to perform experiments varying the flow rate, initial solute concentration, and column height to determine the operating conditions that allow the column to be efficient.

## 4. Materials and Methods

### 4.1. Materials

Food-grade chitosan with a 90% degree of deacetylation was purchased from América Alimentos (Zapopan, México). Magnetite was purchased as Fe(II, III) oxide nanopowder with 50–100 nm particle size (determined by the manufacturer by SEM) from Sigma-Aldrich (Guangzhou, China) and was used as received. Acetic acid and sodium hydroxide were obtained from Fermont (Monterrey, México). Ammonium molybdate tetrahydrate, ascorbic acid, hydrochloric acid, and sulphuric acid were purchased from Golden Bell (Zapopan, México). Potassium antimony(III) tartrate hydrate was purchased from Sigma Aldrich (Bangalore, India). Dibasic sodium arsenate heptahydrate was obtained from Merck (Darmstadt, Germany).

### 4.2. Preparation of ChM Hydrogel Beads

A mass of 22.5 g of powdered chitosan was dissolved in 500 mL of 2% (*v/v*) CH_3_COOH aqueous solution. Then, a mass of 2.25 g of magnetite nanopowder was added to the chitosan solution under stirring using a hand-held processor until a dispersion of homogenous appearance was obtained. ChM hydrogel beads were formed by adding the dispersion dropwise to a 1 M NaOH aqueous solution, using a Masterflex 07557 peristaltic pump with a Masterflex L/S 14 silicone hose. ChM beads were kept for 24 h in the NaOH solution for maturation. Next, the hydrogel beads were washed with bi-distilled water until a pH of 7.0 was obtained in the filtrate. ChM beads were stored in bi-distilled water in refrigeration until used.

### 4.3. Characterization of Hydrogel Beads

#### 4.3.1. Hydrogel Beads Composition

The water content of the ChM hydrogel and the Ch hydrogel were obtained by gravimetric analysis; 1.0 g of beads were dried in a MMM Venticell stove at 60 °C until constant weight (Equation (1)). Two replicas were performed for this procedure for both hydrogel bead types. The magnetite content of a dry sample was determined by gravimetric analysis, since chitosan decomposes at a temperature of 600 °C [32]. To achieve this, dried ChM beads were pulverized using an agate mortar, and a mass of 1.0 g of this powder was placed in a porcelain crucible and burnt at a temperature of 1000 °C for 4 h in a Ney M-525 Series II muffle until the obtention of ashes. This procedure was performed in duplicate. A mass of 1.0 g of chitosan was also burnt at a temperature of 1000 °C in the muffle to determine the ash content of chitosan, and this procedure was also performed in duplicate.

Since the magnetite content determined by the abovementioned gravimetric procedure corresponds to the xerogel, the magnetite content of the hydrogel was obtained by using Equation (2). Equation (3) was used to obtain the chitosan content in the ChM beads.

(1) $$\%H_{2}O=\frac{m_{hydrogel}-m_{xerogel}}{m_{hydrogel}}100\%$$

(2) $$\%{Magnetite}_{hydrogel}=\left(\frac{m_{ChM\;ashes}}{m_{xerogel}}-\frac{m_{chitosan\;ashes}}{m_{xerogel}}\right)\frac{m_{xerogel}}{m_{hyrogel}}100\%$$

(3) $$\%{Chitosan}_{hydrogel}=100\%-\%H_{2}O-\%{Magnetite}_{hydrogel}$$

#### 4.3.2. Average Diameter of Hydrogel Beads

The average diameter of the ChM hydrogel beads and Ch hydrogel beads was obtained by measuring a sample of 30 beads of each type using a digital electronic calibrator.

#### 4.3.3. Morphology and Surface Characterization of the Hydrogel Beads

The morphology and surface of the ChM hydrogel beads were observed on a Hitachi TM 1000 scanning electron microscope operated at an acceleration voltage of 15.0 kV and an emission current of 48 mA.

#### 4.3.4. Potentiometric Titration of ChM

The pKa of Ch and ChM was determined by potentiometric titration following the method reported by Ríos-Donato et al. [34]; a mass of 0.2 g of pulverized Ch or ChM was suspended in 0.1 M HCl and titrated with 0.1 M NaOH. An Ohaus Starter 2100 potentiometer was used for the measurement of pH. This procedure was done in duplicate for Ch and ChM.

#### 4.3.5. FTIR Characterization of ChM

Ch and ChM were characterized by Fourier Transform Infrared Spectroscopy on a FTIR spectrometer (Perkin Elmer, Waltham, MA, USA). A mass of 5 mg of Ch or ChM were pulverized with 100 mg of KBr using an agate mortar and pestle, and then were packed in the sample cup. Samples were read from 4000 to 450 cm^−1^.

#### 4.3.6. ChM-Arsenate Interactions Characterization

The interactions between arsenate and ChM composite were characterized by X-ray Photoelectron Spectroscopy (XPS) using a XPS SPECS System (Berlin, Germany), which contains a Phoibos analyzer and a 1D DLD detector. The XPS spectra were obtained with a monochromatic Al Kα source (1486.6 eV) working at 250 W (12.5 kV and 20 mA) and a base pressure of 3 × 10^−9^ mbar in the analytical chamber. The high-resolution scans were conducted with a pass energy of 15 eV energy to compensate. Data were analyzed with the Analyzer 2.21 software, using Lorentzian–Gaussian curves after background subtraction.

### 4.4. Fixed Bed Column Studies on Arsenate Iones Adsorption from Aqueous Solution

The fixed-bead columns adsorption of arsenate ions was carried out at 25 °C. Glass columns were used with a bed height of 13 or 33 cm, with an internal diameter of 1.8 cm, and packed with 17.2 or 43.7 g of the Ch or ChM hydrogel beads, respectively. The volumes of the mobile phase in the spaces between the beads in the column, also referred to as interstitial bed volume (IVB), were 10.8 mL and 27.4 mL for the 13 and 33 cm height columns respectively. To feed the column with the arsenate ion solution, a Masterflex 07557 peristaltic pump with Masterflex L/S 14 silicone hoses was used. A solution with a chosen predetermined concentration of arsenate ions (2, 5 or 10 mg As/L) was fed to the bottom of the column at the desired flow rate (13, 20 or 25 mL/h) regulated by the peristaltic pump. The flow direction was from the bottom to the top of the column to avoid channeling of the influent solution [62,63]. Effluent samples were collected at different time intervals. The amount of As(V) remaining in the solution was determined using the molybdenum blue method [64]. The pH of the samples was measured using an Ohaus 2100 Starter potentiometer. Each adsorption of arsenate ions by fixed bed columns was done in duplicate.

The portion of protonated and unprotonated amine groups of ChM at a given pH value was obtained by the Henderson–Hasselbach equation (Equation (4)), using the pKa value determined by potentiometric titration.

(4) $$pH={pK}_{a}+log\frac{\left[-NH_{2}\right]}{\left[-{NH}_{3}^{+}\right]}$$

### 4.5. Fixed Bed Column Data Analysis

The adsorption capacity of the columns was obtained from the concentration profile of the solution at the column exit. This concentration profile is referred to as the breakthrough curve and is commonly expressed as the dimensionless concentration C_t_/C_0_ as a function of time, volume, or the number of interstitial bed volumes (NIVB). C_t_ is the As(V) concentration of the solution at a given NIBV, while C_0_ is the concentration of the feed solution. Equation (5) was used to determine column capacity at the breakthrough point (q_b_) and Equation (6) was used for the estimation of column capacity at exhaustion (q_e_). Time t_b_ is referred to as the breakthrough time and is attained when the concentration of the effluent C_t_ reaches the maximum allowed percentage of the feed solution, and its value is usually taken as 5% of C_0_ [18,19,65,66]; for the present work, NIBV at t_b_ was assigned when C_t_ reached 5% of C_0_. Column exhaustion capacity was determined when C_t_ reached 95% of C_0_. Q represents the volumetric flow rate and m is the dry mass of the hydrogel beads in Equations (4) and (5).

(5) $$q_{b}=\frac{QC_{0}}{m}\int_{0}^{NIVB\;at\;t_{b}}\left(1-\frac{C_{t}}{C_{0}}\right)dt$$

(6) $$q_{e}=\frac{QC_{0}}{m}\int_{0}^{NIVB\;at\;t_{e}}\left(1-\frac{C_{t}}{C_{0}}\right)dt$$

Although the column operation stops once the breakthrough point is reached for large-scale water treatment, for the design of adsorption columns, the breakthrough curve is obtained using small-scale columns [18,44,46,67]. The lengths of the used (H_L_) and unused bed length (H_LUB_) can be obtained by Equation (7) and Equation (8), respectively, where the ratio q_b_/q_e_ is the fraction of the adsorbent used for removing arsenate up at the breakthrough point, and H represents the total length of the column. The H_LUB_ represents the mass transfer zone (MTZ) where the adsorption of adsorbate takes place during the column operation [19,44,46,67]. The ratio H_L_/H_LUB_ was used to define the efficiency of the column.

(7) $$H_{L}=H\frac{q_{b}}{q_{e}}$$

(8) $$H_{LUB}=H\left(1-\frac{q_{b}}{q_{e}}\right)$$

### 4.6. Fixed Bed Models

Experimental data of the MTZ region of the breakthrough curve obtained for each fixed bed treatment were fitted to the Thomas, Yoon–Nelson, and Bohart–Adams models. Since adsorption models are non-linear, the estimation of the models’ parameters was done by non-linear regression analysis using Origin Pro 2016 software; by doing so, the error function obtained was appropriate for the estimation of such parameters [68,69].

The Thomas model is the most widely used model reported in the literature for fixed-bed column studies [69,70]. This model is useful for the estimation of the fixed-bed’s adsorption capacity and for the prediction of the breakthrough curves [71]. The Thomas model was proposed for liquid-solid adsorption under the supposition of a Langmuir isotherm, reversible second-order kinetics, and that the resistances to internal and external diffusion are negligible [69,71]. This model does not take into account axial dispersion [72]. Equation (9) represents the Thomas model, where k_Th_ is the Thomas constant and q_Th_ is the fixed bed’s uptake capacity [73].

(9) $$\frac{C_{t}}{C_{0}}=\frac{1}{1+\exp\left(\frac{k_{Th}q_{Th}m}{Q}-k_{Th}C_{0}t\right)}$$

The Yoon–Nelson model is a simple model that assumes that axial dispersion is negligible and was proposed for gas-solid adsorption. [74]. However, it is also used in the literature for the analysis of liquid-solid systems [72]. This model allows obtaining the time required to reach 50% of the breakthrough curve [74,75]. Equation (10) represents the Yoon–Nelson model, where k_YN_ is the Yoon–Nelson parameter, and τ is the contact time required to reach 50% of the adsorbent’s saturation.

(10) $$\frac{C}{C_{0}}=\frac{\exp\left(k_{YN}t-k_{YN}\tau \right)}{1+exp\left(k_{YN}t-k_{YN}\tau \right)}$$

The Bohart–Adams model makes the supposition that the rate at which the adsorption occurs is proportional to both the residual uptake capacity of the adsorbent and the residual solute concentration [72,76]. This model considers axial dispersion to be insignificant. Equation (11) represents the Bohart–Adams model; k_BA_ is the model’s kinetic parameter, N_0_ is the maximum adsorption capacity, and u is the linear velocity of the fluid.

(11) $$\frac{C}{C_{0}}=\exp\left(k_{BA}C_{0}t-k_{BA}N_{0}\frac{H}{u}\right)$$

## Figures and Tables

**Table 1:** Results obtained in the arsenate adsorption at pH 7.0 using fixed-bed columns packed with ChM or Ch hydrogel beads.

Adsorbent	C_0_ (mg/L)	Q (mL/h)	H (cm)	H_LUB_ (cm)	H_L_/H_LUB_	V (mL) at t_b_	NIVB at t_b_	NIVB at t_e_	q_b_ (mg/g)	q_e_ (mg/g)
ChM	5	13	13	7.78 ± 0.26	0.67 ± 0.06	112.4 ± 4.3	10.4 ± 0.4	57.1 ± 1.2	0.48 ± 0.02	1.19 ± 0.10
	5	20	13	9.54 ± 0.60	0.36 ± 0.08	70.2 ± 4.9	6.5 ± 0.5	68.9 ± 3.5	0.30 ± 0.02	1.15 ± 0.12
	5	25	13	9.75 ± 0.95	0.33 ± 0.19	73.3 ± 9.8	6.8 ± 1.0	59.0 ± 4.8	0.31 ± 0.07	1.12 ± 0.08
	2	13	33	6.60 ± 0.02	4.00 ± 0.01	1564.1 ± 43.3	57.1 ± 1.6	163.3 ± 8.0	2.70 ± 0.08	3.37 ± 0.10
	5	13	33	10.19 ± 0.46	2.24 ± 0.14	440.9 ± 8.2	16.1 ± 0.3	123.0 ± 0.0	2.18 ± 0.04	3.15 ± 0.00
	10	13	33	11.95 ± 0.08	2.12± 0.02	264.6 ± 9.0	9.7 ± 0.3	60.9 ± 3.4	2.61 ± 0.09	3.84 ± 0.12
Ch	2	13	33	31.90 ± 0.03	0.03 ± 0.00	34.1 ± 3.8	1.2 ± 0.1	86.2 ± 13.2	0.02 ± 0.00	0.60 ± 0.08
	5	13	33	29.57 ± 0.01	0.12 ± 0.00	78.6 ± 7.2	2.9 ± 0.3	90.4 ± 7.5	0.10 ± 0.01	0.95 ± 0.08
	10	13	33	27.45 ± 0.30	0.20 ± 0.01	82.6 ± 1.7	3.0 ± 0.1	32.4 ± 2.3	0.21 ± 0.00	1.24 ± 0.09

**Table 2:** Adsorption capacity values reported for the removal of As(V) at pH near 7 using different iron-containing adsorbent materials in fixed bed operations.

Adsorbent	q_e_ (mg As/g)	Reference
Iron oxide-coated alginate	0.023	[49]
Iron crosslinked alginate	0.066	[50]
Iron-impregnated *Azadirachta indica* biochar	0.149	[51]
Natural laterite	0.20	[52]
Iron-modified biochar	0.303	[53]
Pectin stabilized nano zero valent iron particles	0.318	[54]
Iron-mixed mesoporous pellet	0.352	[55]
Hydrated mixed trivalent iron-aluminum oxide	0.569	[56]
Iron-coated calcined bauxite	0.606	[57]
Iron-coated cork granulates	2.0	[27]
Chitosan-magnetite hydrogel beads	3.84	This study

**Table 3:** Parameters of fixed-bed models (Thomas, Yoon–Nelson, and Bohart–Adams) obtained by non-linear regression analysis.

Adsorbent	ChM	ChM	ChM	ChM	ChM	ChM	Ch	Ch	Ch
H $\left(cm\right)$	13	13	13	33	33	33	33	33	33
C_0_ $\left(\frac{mg}{L}\right)$	5	5	5	2	5	10	2	5	10
Q $\left(\frac{mL}{h}\right)$	13	20	25	13	13	13	13	13	13
Thomas									
k_Th_ $\left(\frac{L}{mg\cdot h}\right)$	4.75 × 10^−4^	7.05 × 10^−4^	7.13 × 10^−4^	3.17 × 10^−4^	4.45 × 10^−4^	2.74 × 10^−4^	5.50 × 10^−4^	7.79 × 10^−4^	2.66 × 10^−4^
q_Th_ $\left(\frac{mg}{g}\right)$	104.0	82.4	86.6	35.8	45.4	59.5	21.7	35.5	47.9
R^2^	0.9956	0.9349	0.9851	0.9516	0.9440	0.7991	0.9829	0.9804	0.9830
MAE	0.0228	0.0683	0.0277	0.0502	0.0477	0.1174	0.0360	0.0388	0.0315
Yoon–Nelson									
k_YN_ $\left(\frac{1}{min}\right)$	3.54 × 10^−3^	5.35 × 10^−3^	5.30 × 10^−3^	3.32 × 10^−4^	1.33 × 10^−3^	1.63 × 10^−3^	6.40 × 10^−4^	1.96 × 10^−3^	1.40 × 10^−3^
τ $\left(min\right)$	1152.5	627.7	531.5	7720.4	3340.2	2215.3	4196.5	3217.9	2160.2
R^2^	0.9891	0.9349	0.9851	0.9516	0.9440	0.7991	0.9829	0.9804	0.9830
MAE	0.0196	0.0683	0.0277	0.0503	0.0477	0.1175	0.0360	0.0387	0.0315
Adams–Bohart									
k_BA_ $\left(\frac{L}{mg\cdot min}\right)$	8.49 × 10^−5^	7.01 × 10^−5^	2.40 × 10^−4^	7.52 × 10^−5^	6.71 × 10^−6^	1.56 × 10^−5^	9.53 × 10^−5^	7.28 × 10^−5^	7.85 × 10^−5^
N_0_ $\left(\frac{mg}{L}\right)$	105.8	173.2	114.4	83.7	593.2	215.5	71.0	85.3	113.0
R^2^	0.5850	0.6120	0.8610	0.8769	0.6203	0.4773	89.5613	0.7471	0.9505
MAE	0.1382	0.1654	0.0902	0.1036	0.1094	0.1737	0.0875	1.1954	0.0607

## Data Availability

Not applicable.
